# Higher Meat Intake Is Associated with Higher Inflammatory Markers, Mostly Due to Adiposity: Results from UK Biobank

**DOI:** 10.1093/jn/nxab314

**Published:** 2021-09-29

**Authors:** Keren Papier, Lilian Hartman, Tammy Y N Tong, Timothy J Key, Anika Knuppel

**Affiliations:** Cancer Epidemiology Unit, Nuffield Department of Population Health, University of Oxford, Oxford, United Kingdom; John Radcliffe Hospital, Medical Sciences Division, University of Oxford, Oxford, United Kingdom; Lincoln College, University of Oxford, Oxford, United Kingdom; Cancer Epidemiology Unit, Nuffield Department of Population Health, University of Oxford, Oxford, United Kingdom; Cancer Epidemiology Unit, Nuffield Department of Population Health, University of Oxford, Oxford, United Kingdom; Cancer Epidemiology Unit, Nuffield Department of Population Health, University of Oxford, Oxford, United Kingdom

**Keywords:** inflammation, meat intake, cohort study, UK Biobank, C-reactive protein, white blood cell count

## Abstract

**Background:**

High meat consumption might play a role in promoting low-grade systemic inflammation, but evidence is limited.

**Objectives:**

We examined cross-sectional associations of habitual meat consumption with serum C-reactive protein (CRP) and total white blood cell count (WBCC) in British adults.

**Methods:**

We included 403,886 men and women (aged 38–73 y) participating in the UK Biobank who provided information on meat intake (via touchscreen questionnaire) and a nonfasting blood sample at recruitment (2006–2010). For a subset of participants (∼5%), an additional blood sample was collected (median 4.4 y later). We used multivariable linear regression models to estimate associations of meat intake (total meat, unprocessed red meat, processed meat, and poultry) with logCRP and logWBCC.

**Results:**

The difference in the serum CRP (mg/L) for each 50-g/d higher intake for total meat was 11.6% (95% CI: 11.1, 12.0%), for processed meat was 38.3% (95% CI: 36.0, 40.7%), for unprocessed red meat was 14.4% (95% CI: 13.6, 15.1%), and for poultry was 12.8% (95% CI: 12.0, 13.5%). The difference in the WBCC (×10^–9^L) for each 50 g/d higher intake of total meat was 1.5% (95% CI: 1.4, 1.6%), for processed meat was 6.5% (95% CI: 6.1, 6.9%), for unprocessed red meat was 1.6% (95% CI: 1.4, 1.7%), and for poultry was 1.6% (95% CI: 1.4, 1.7%). All associations were attenuated after adjustment for adiposity; by 67% with BMI (in kg/m^2^) and by 58% with waist circumference for total meat and CRP, and by 53% and 47%, respectively, for WBCC, although associations remained statistically significant. Findings of sensitivity analyses in 15,420 participants were similar prospectively, except there were no associations between unprocessed red meat and WBCC.

**Conclusions:**

Higher meat consumption, particularly of processed meat, was positively associated with inflammatory markers in these British adults; however, the magnitudes of associations are small and predominantly due to higher adiposity.

## Introduction

Systemic low-grade inflammation, characterized by increases in inflammatory biomarkers such as C-reactive protein (CRP), white blood cell count (WBCC), interleukin 6 (IL-6), and Tumour Necrosis Factor alpha (TNF-α) (1), has been associated with a higher risk of some chronic diseases such as type 2 diabetes (2) and all-cause mortality (3). It has been suggested that high meat consumption might play a role in inflammatory processes, possibly through its high amounts of heme iron (4), saturated fat content (5), and advanced glycation end products (AGEs) (6). Another possibility is that an association of meat and inflammation is confounded or mediated by increased adiposity (central or general), which has been found to be related to meat intake (7) and inflammation, with genetic evidence suggesting that the relation of adiposity and inflammation is causal (8).

The available evidence for associations of meat intake with markers of systemic inflammation is inconsistent, based on small studies (<17 k), and mostly focused on red meat. Most (9–13) but not all previous studies (14, 15) have found a positive association between red meat (9–13), processed meat (11, 12, 15), and CRP before adjustment for adiposity, and no association with poultry (15). Of the studies that adjusted for adiposity, most (10–13) but not all (9) reported that the association of meat with CRP was no longer significant, suggesting that the association may be due to higher adiposity.

The aim of the current study was to assess the associations of habitual consumption of different types of meat (including total meat, unprocessed red meat, processed meat, and poultry) with CRP and WBCC in a large cohort of British adults, and to clarify the role of adiposity.

## Methods

### Study population

This cross-sectional study was based on 403,886 men and women aged between 38 and 73 y, registered with the National Health Service in England, Wales, and Scotland, and enrolled in the UK Biobank cohort study between 2006 and 2010 (16). The study was conducted in accordance with the Helsinki Declaration of 1975 as revised in 1983, and approved by the National Information Governance Board for Health and Social Care and the National Health Service North West Multicentre Research Ethics Committee (16/NW/0274), and participants provided informed consent.

### Exposure, outcome, and covariate collection

Usual dietary intake was collected at recruitment using a touchscreen questionnaire that included 29 questions on diet, assessing the consumption frequency of each listed food (as of 22 July 2021; https://biobank.ctsu.ox.ac.uk/crystal/crystal/docs/TouchscreenQuestionsMainFinal.pdf). Unprocessed red meat was defined as the sum of the responses to 3 questions on red meat, which included beef, lamb/mutton, and pork, while processed meat (e.g. bacon, ham, sausages, meat pies, kebabs, burgers, chicken nuggets), and poultry intake (including chicken, turkey, or other poultry) were based on 1 question each. To investigate the combined effects, all meat types were summed as total meat intake. Meat intakes were categorized into groups based on weekly intake frequency depending on data distribution, as reported previously (17). We calculated meat intake in grams by assigning a portion size of 120 g for unprocessed red meat, 50 g for processed meat, and 130 g for poultry (18).

All participants provided a nonfasting blood sample at recruitment and a subsample of participants (*n* = 20,345; 21% of those invited) who lived within a 35-km radius of the UK Biobank Co-ordinating Centre in Stockport, England, provided an additional nonfasting blood sample a median of 4.4 y later (min 2.1 y, max 7.0 y) between 2012 and 2013 (as of 25 May, 2021; https://biobank.ctsu.ox.ac.uk/∼bbdatan/Repeat_assessment_doc_v1.0.pdf). Samples were subsequently kept at 4°C during shipping to the purpose-built laboratory for UK Biobank in Stockport, England (as of 25 May 2021; https://biobank.ndph.ox.ac.uk/∼bbdatan/biomarkers.pdf); complete blood cell counts (including WBCC in ×10^–9^ cells/L) were conducted within 24 h of venipuncture using a Coulter Counter (Beckman Coulter), and serum CRP concentrations (mg/L) were measured later in stored samples using high-sensitivity immunoturbidimetry. The average within-laboratory coefficients of variation (ratio of the SD to the mean) for CRP were 2.31% for low concentrations, 1.70% for medium concentrations, and 1.69% for high concentrations (as of 25 May 2021; https://biobank.ndph.ox.ac.uk/showcase/showcase/docs/serum_biochemistry.pdf).

All covariates except for waist circumference, weight, and height were ascertained via the touchscreen questionnaire. Waist circumference was measured using a Wessex nonstretchable sprung tape (passed around the smallest part of the trunk (i.e., the natural indent) or the umbilicus if the natural indent was not found), height using a stadiometer, and weight using a Tanita BC418MA body composition analyzer to perform part of a Bioimpedance Analysis (BIA) or a standard scale in participants that did not participate in BIA. All measurements were conducted by trained staff according to standard procedures. (as of 15 September 2021; https://biobank.ndph.ox.ac.uk/ukb/ukb/docs/Anthropometry.pdf).

For all analyses, study participants were excluded if they had withdrawn from the study (*n* = 829), had missing data on CRP and WBCC at baseline (*n* = 45,965), or missing data on meat intake (*n* = 6806) or any covariates (*n* = 45,831) resulting in an analytical sample of 403,886 participants (**Supplemental Figure 1**).

### Statistical analysis

We used multivariable linear regression models to investigate the associations of habitual meat intake with log(CRP) and log(WBCC); CRP and WBCC were logarithmically transformed to satisfy model assumptions and normalize distributions. For trend analyses per 50 g/d higher intake, β coefficients were exponentiated to yield percentage differences and corresponding 95% CIs. For categorical meat intakes, associations were expressed as geometric means with 95% CIs using the margins postestimation command in Stata. We used 4 models to assess the effects of potential confounders (models 1 and 2) and adiposity (models 3 and 4) on observed associations. In model 1, adjustments were made for age and sex, and model 2 was additionally adjusted for baseline smoking status, ethnicity, Townsend deprivation index, employment, qualification level, total fruit and vegetable intake, fiber intake from bread and breakfast cereals, total fish consumption, total physical activity, alcohol intake, and menopausal status in women. In models 3 and 4, we additionally adjusted the full model (model 2) for BMI (in kg/m^2^) and waist circumference (in cm), respectively.

The relation between meat intake and inflammation may vary by sex (12). Therefore, we assessed heterogeneity by sex in the associations of meat intake (per 50 g/d higher intake) with logCRP and logWBCC by adding an interaction term to test for statistical significance using likelihood ratio tests and by stratifying results. We conducted sensitivity analyses in a subsample of 15,420 adults with serum biomarker measures at follow-up, 4.4 (median) y after baseline. All analyses were performed using Stata Release 16.1, StataCorp LLC.

## Results

**Table 1** shows characteristics of the analytical sample and participants by categories of total meat intake. With higher meat intake the proportions of participants who were men, former or current smokers, less physically active, or consumed more alcohol were higher, and intakes of fruit and vegetables and cereal fiber were lower. Furthermore the proportions of participants of white European ethnicity, who were affluent, had a lower level of education, and who were retired were higher in the highest (≥7 times/wk) meat intake category than in the lowest (<3 times/wk) meat intake category but there was no clear trend across categories. Participants in the highest (≥7 times/wk) meat intake category had a 2.1 kg/m^2^ higher mean BMI and 5.2 cm higher waist circumference compared with participants who reported the lowest meat intakes (<3 times/wk), with a trend across meat intake categories.

**FIGURE 1:**
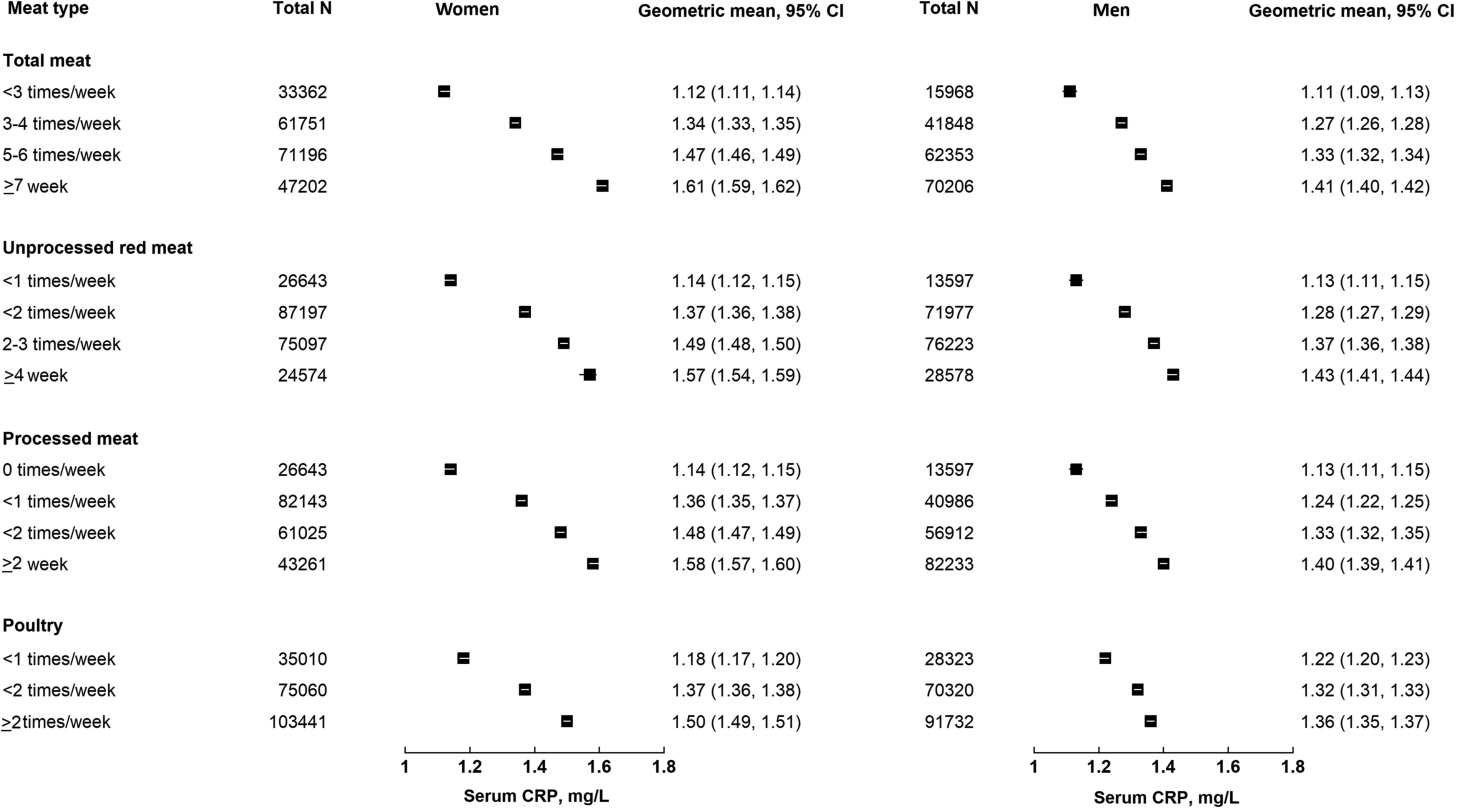
Adjusted geometric means of serum CRP (mg/L) and 95% CI by meat types and sex. Adjusted for age, baseline smoking status (never, former, current smoker <15 cigarettes/d, ≥15 cigarettes/d, unknown amount), ethnicity (white, nonwhite), Townsend deprivation index (quintiles from least to most deprived), employment (employed or self-employed, retired, unemployed), and qualification level (college or university degree or vocational qualification, national examination at ages 17–18 y, national examination at age 16 y, other or unknown), total fruit and vegetable intake (<3, 3–3.99, 4–5.99, ≥6 servings/d), bread and cereal fiber intake (sex-specific quintiles), total fish consumption (0–1, >1 to <2, 2 to <3, ≥3 times/wk), total physical activity (<5, 5–9.9, 10–14.9, 15–24.9, 25–34.9, 35–49.9, 50–74.9, 75–99.9, ≥100 MET h/wk), alcohol intake (<1, 1 to <5, 5 to <10, 10 to <15, 15 to <20, 20 to <25, ≥ 25, nondrinkers) and menopausal status (premenopausal, postmenopausal) in women. CRP, C-reactive protein; MET, metabolic equivalent.

**TABLE 1:** Characteristics of the analytical sample by total meat intake frequency^1^

		Total meat intake frequency			
Characteristics	Analytical sample (*n* = 403,886)	<3 times/wk (*n* = 49,330)	3 to <5 times/wk (*n* = 103,599)	5 to <7 times/wk (*n* = 133,549)	≥7 times/wk (*n* = 117,408)
Sex					
Women	213,511 (52.9)	33,362 (67.6)	61,751 (59.6)	71,196 (53.3)	47,202 (40.2)
Men	190,375 (47.1)	15,968 (32.4)	41,848 (40.4)	62,353 (46.7)	70,206 (59.8)
Age, y	56.7 ± 8.1	55.9 ± 8.1	57.6 ± 7.9	56.9 ± 8.0	56.1 ± 8.2
Ethnicity					
White	385,694 (95.5)	44,986 (91.2)	99,617 (96.2)	129,136 (96.7)	111,955 (95.4)
Nonwhite	18,192 (4.5)	4344 (8.8)	3982 (3.8)	4413 (3.3)	5453 (4.6)
Townsend deprivation					
Most affluent	84,218 (20.9)	8291 (16.8)	22,101 (21.3)	29,413 (22.0)	24,413 (20.8)
Most deprived	74,431 (18.4)	11,445 (23.2)	18,123 (17.5)	22,390 (16.8)	22,473 (19.1)
Qualification					
College or university degree/vocational qualification	247,738 (61.3)	32,989 (66.9)	62,517 (60.3)	80,382 (60.2)	71,850 (61.2)
National examination at ages 17–18 y	22,617 (5.6)	2727 (5.5)	5519 (5.3)	7540 (5.6)	6831 (5.8)
National examination at age 16 y	67,283 (16.7)	6,895 (14.0)	17,266 (16.7)	23,433 (17.5)	19,689 (16.8)
Other/unknown	66,248 (16.4)	6719 (13.6)	18,297 (17.7)	22,194 (16.6)	19,038 (16.2)
Employment					
In paid employment	233,897 (57.9)	30,486 (61.8)	56,895 (54.9)	76,288 (57.1)	70,228 (59.8)
Retired	127,058 (31.5)	13,116 (26.6)	36,183 (34.9)	43,632 (32.7)	34,127 (29.1)
Not in paid employment	42,931 (10.6)	5728 (11.6)	10,521 (10.2)	13,629 (10.2)	13,053 (11.1)
Smoking					
None	221,188 (54.8)	28,493 (57.8)	57,738 (55.7)	73,557 (55.1)	61,400 (52.3)
Former	142,320 (35.2)	16,556 (33.6)	36,386 (35.1)	47,479 (35.6)	41,899 (35.7)
Current <15 cigarettes/d	11,593 (2.9)	1462 (3.0)	2887 (2.8)	3640 (2.7)	3604 (3.1)
Current ≥15 cigarettes/d	15,532 (3.8)	1306 (2.6)	3344 (3.2)	4760 (3.6)	6122 (5.2)
Current, unknown amount	13,253 (3.3)	1513 (3.1)	3244 (3.1)	4113 (3.1)	4383 (3.7)
Physical activity level, MET h/wk					
<5	50,223 (12.4)	5485 (11.1)	12,829 (12.4)	16,706 (12.5)	15,203 (12.9)
≥100	40,603 (10.1)	4904 (9.9)	9825 (9.5)	12,859 (9.6)	13,015 (11.1)
Alcohol intake					
<1 g/d	43,135 (10.7)	7166 (14.5)	11,788 (11.4)	13,745 (10.3)	10,436 (8.9)
≥25 g/d	81,306 (20.1)	5471 (11.1)	16,450 (15.9)	26,532 (19.9)	32,853 (28.0)
Non-drinkers	29,758 (7.4)	6524 (13.2)	7842 (7.6)	8122 (6.1)	7270 (6.2)
Fruit and vegetable intake, servings^2^/d	4.7 ± 2.6	5.4 ± 3.0	4.7 ± 2.5	4.6 ± 2.4	4.5 ± 2.5
Estimated cereal fiber intake, g/d	4.6 ± 2.9	4.7 ± 3.1	4.6 ± 2.9	4.6 ± 2.9	4.5 ± 3.0
Fish intake, times/wk					
0–1	101,960 (25.2)	18,485 (37.5)	23,676 (22.9)	30,026 (22.5)	29,773 (25.4)
<2	88,992 (22.0)	7115 (14.4)	23,952 (23.1)	30,890 (23.1)	27,035 (23.0)
<3	96,942 (24.0)	7950 (16.1)	27,020 (26.1)	34,284 (25.7)	27,688 (23.6)
≥3	115,992 (28.7)	15,780 (32.0)	28,951 (27.9)	38,349 (28.7)	32,912 (28.0)
Menopausal status					
Premenopausal	52,284 (24.5)	9041 (27.1)	12,968 (21.0)	17,215 (24.2)	13,060 (27.7)
Postmenopausal	161,227 (75.5)	24,321 (72.9)	48,783 (79.0)	53,981 (75.8)	34,142 (72.3)
BMI^3^, kg/m^2^	27.3 (27.3, 27.3)	26.0 (26.0, 26.1)	26.9 (26.9, 27.0)	27.5 (27.5, 27.5)	28.1 (28.0, 28.1)
Waist circumference^3^, cm	90.2 (90.2, 90.3)	87.2 (87.1, 87.4)	89.2 (89.2, 89.3)	90.0 (90.5, 90.6)	92.0 (92.0, 92.1)

1 Values are presented as *n* (%) of participants, means (95% CIs), or means ± SDs. All associations *P* < 0.001 based on ANOVA for characteristics presented as means ± SDs and Pearson's chi for those presented as *n* (%).

2 Each serving of fruit and vegetable is equivalent to 1 piece of fresh fruit (approximately 80 g), 2 pieces of dried fruit (approximately 15 g) or 2 heaped tablespoons of vegetables (approximately 50 g) (28).

3 Arithmatic means adjusted for sex and age.

In multivariable adjusted models (model 2), each additional 50 g/d intake of meat was associated with higher CRP. The difference in the serum CRP (mg/L) for each 50 g/d higher intake of total meat was 11.6% (95% CI: 11.1, 12.0%), of processed meat was 38.3% (95% CI: 36.0, 40.7%), of unprocessed red meat was 14.4% (95% CI: 13.6, 15.1%), and of poultry was 12.8% (95% CI: 12.0, 13.5%). (**Table 2**). There were significant interactions by sex for all associations with CRP (*P* < 0.001). In stratified results both women and men showed positive associations, with larger associations observed in women [the difference in the serum CRP (mg/L) for each 50 g/d higher intake of total meat in women: 15.2% (95% CI: 14.5,15.9%), in men: 7.9% (95% CI:7.4,8.5%); see Table 2 for meat subtypes). **Figure 1** shows geometric means for CRP in women and men by categories of meat intakes (based on model 2).

**TABLE 2:** Mean percentage difference (95% CI) in serum CRP and WBCC per 50 g/d higher meat intake by level of adjustment (women, *n* = 213,511; men, *n* = 190,375)^1^

	Model 1^2^		Model 2^3^		Model 2 + BMI^4^			Model 2 + WC^5^		
Meat type per 50 g/d	Difference, % (95% CI)	*P*-heterogeneity^6^	Difference, % (95% CI)	*P*-heterogeneity^6^	Difference, % (95% CI)	*P*-heterogeneity^6^	Change due to BMI, %^7^	Mean change, % (95% CI)	*P*-heterogeneity^6^	Change due to WC, %^7^
Serum CRP, mg/L										
Total meat^8^	12.6 (12.1, 13.0)	<0.001	11.6 (11.1, 12.0)	<0.001	3.8 (3.5, 4.2)	<0.001	67.2	4.9 (4.5, 5.2)	<0.001	57.8
Women	15.4 (14.7, 16.0)		15.2 (14.5, 15.9)		4.7 (4.2, 5.2)		69.1	6.1 (5.5, 6.6)		59.9
Men	9.7 (9.1, 10.3)		7.9 (7.4, 8.5)		2.9 (2.4, 3.5)		63.3	3.4 (2.9, 3.9)		57.0
Unprocessed red meat^8^	16.9 (16.1, 17.6)	<0.001	14.4 (13.6, 15.1)	<0.001	5.7 (5.1, 6.4)	<0.001	60.4	6.6 (5.9, 7.2)	<0.001	54.2
Women	18.9 (17.7, 20.1)		18.4 (17.3, 19.5)		6.8 (5.9, 7.7)		63.0	8.1 (7.2, 9.1)		56.0
Men	14.9 (13.9, 15.9)		10.6 (9.6, 11.5)		4.7 (3.9, 5.6)		55.7	5.0 (4.1, 5.8)		52.8
Processed meat^8^	53.2 (50.7, 55.8)	<0.001	38.3 (36.0, 40.7)	<0.001	14.6 (12.8, 16.4)	<0.001	61.9	13.1 (11.3, 14.8)	<0.001	65.8
Women	69.5 (65.0, 74.3)		56.7 (52.5, 61.0)		15.9 (13.1, 18.7)		72.0	15.9 (13.1, 18.7)		72.0
Men	43.0 (40.0, 46.0)		25.9 (23.3, 28.5)		13.2 (11.0, 15.5)		49.0	10.7 (8.5, 12.9)		58.7
Poultry^8^	11.1 (10.3, 11.8)	<0.001	12.8 (12.0, 13.5)	<0.001	2.5 (1.9, 3.2)	<0.001	80.5	4.9 (4.2, 5.5)	<0.001	61.7
Women	17.2 (16.0, 18.3)		18.2 (17.1, 19.4)		4.3 (3.4, 5.2)		76.4	6.8 (5.9, 7.7)		62.6
Men	4.1 (3.0, 5.1)		6.9 (5.8, 7.9)		0.6 (−0.3, 1.6)		91.3	2.3 (1.3, 3.2)		66.7
WBCC, ×10^9^ cells/L										
Total meat^8^	1.6 (1.5, 1.7)	<0.001	1.5 (1.4, 1.6)	<0.001	0.7 (0.6, 0.8)	0.168	53.3	0.8 (0.7, 0.9)	0.037	46.7
Women	1.8 (1.7, 2.0)		1.7 (1.6, 1.9)		0.8 (0.7, 0.9)		52.9	0.9 (0.7, 1.0)		47.1
Men	1.5 (1.4, 1.7)		1.2 (1.1, 1.4)		0.6 (0.5, 0.7)		50.0	0.6 (0.5, 0.8)		50.0
Unprocessed red meat^8^	2.1 (1.9, 2.3)	0.015	1.6 (1.4, 1.7)	0.041	0.7 (0.6, 0.9)	0.707	56.3	0.7 (0.6, 0.9)	0.333	56.3
Women	2.1 (1.8, 2.3)		1.8 (1.6, 2.1)		0.8 (0.6, 1.0)		55.6	0.9 (0.6, 1.1)		50.0
Men	2.2 (2.0, 2.4)		1.3 (1.1, 1.5)		0.6 (0.4, 0.8)		53.8	0.6 (0.4, 0.8)		53.8
Processed meat^8^	9.5 (9.0, 9.9)	<0.001	6.5 (6.1, 6.9)	<0.001	4.4 (4.0, 4.8)	0.039	32.3	4.1 (3.7, 4.5)	0.005	36.9
Women	10.6 (9.9, 11.3)		8.0 (7.3, 8.7)		4.9 (4.3, 5.6)		38.8	4.7 (4.1, 5.4)		41.3
Men	8.9 (8.3, 9.5)		5.4 (4.9, 6.0)		4.0 (3.5, 4.5)		25.9	3.6 (3.1, 4.2)		33.3
Poultry^8^	1.0 (0.8, 1.1)	<0.001	1.6 (1.4, 1.7)	<0.001	0.5 (0.4, 0.7)	0.003	68.8	0.7 (0.6, 0.9)	0.003	56.3
Women	1.7 (1.5, 2.0)		2.0 (1.8, 2.2)		0.8 (0.6, 1.0)		60.0	0.9 (0.7, 1.2)		55.0
Men	0.2 (0.0, 0.5)		1.1 (0.9, 1.3)		0.3 (0.1, 0.6)		72.7	0.5 (0.3, 0.8)		54.5

1 BMI, Body mass index; CRP, C-reactive protein; MET, metabolic equivalent; WBCC, white blood cell count; WC, waist circumference.

2 Model 1 adjusted for age. The percentage difference refers to an increase of in CRP/WBCC for every 50 g/d higher meat intake.

3 Model 2: model 1 + baseline smoking status (never, former, current smoker < 15 cigarettes/d, ≥15 cigarettes/d, unknown amount), ethnicity (nonwhite), Townsend deprivation index (quintiles from least to most deprived), employment (employed or self-employed, retired, unemployed) and qualification level (college or university degree or vocational qualification, national examination at ages 17–18, national examination at age 16, other or unknown), total fruit and vegetable intake (<3, 3–3.99, 4–5.99, ≥6 servings/d), bread and cereal fiber intake (sex-specific quintiles), total fish consumption (0–1, >1 to <2, 2 to <3, ≥3 times/wk), total physical activity (<5, 5–9.9, 10–14.9, 15–24.9, 25–34.9, 35–49.9, 50–74.9, 75–99.9, ≥100 metabolic equivalent h/wk), alcohol intake (<1, 1 < 5, 5 < 10, 10 < 15, 15 < 20, 20 < 25, ≥25, nondrinkers) and menopausal status (premenopausal/postmenopausal) in women.

4 Model 2 + baseline BMI (continuous).

5 Model 2 + baseline WC (continuous).

6 *P*-heterogeneity based on a likelihood-ratio test comparing the model with and without an interaction for sex.

7 BMI/WC percentage change is the proportion of the main association (model 2) attenuated after adjustment for adiposity.

8 Association for women and men combined, all models additionally adjusted for sex.

**FIGURE 2:**
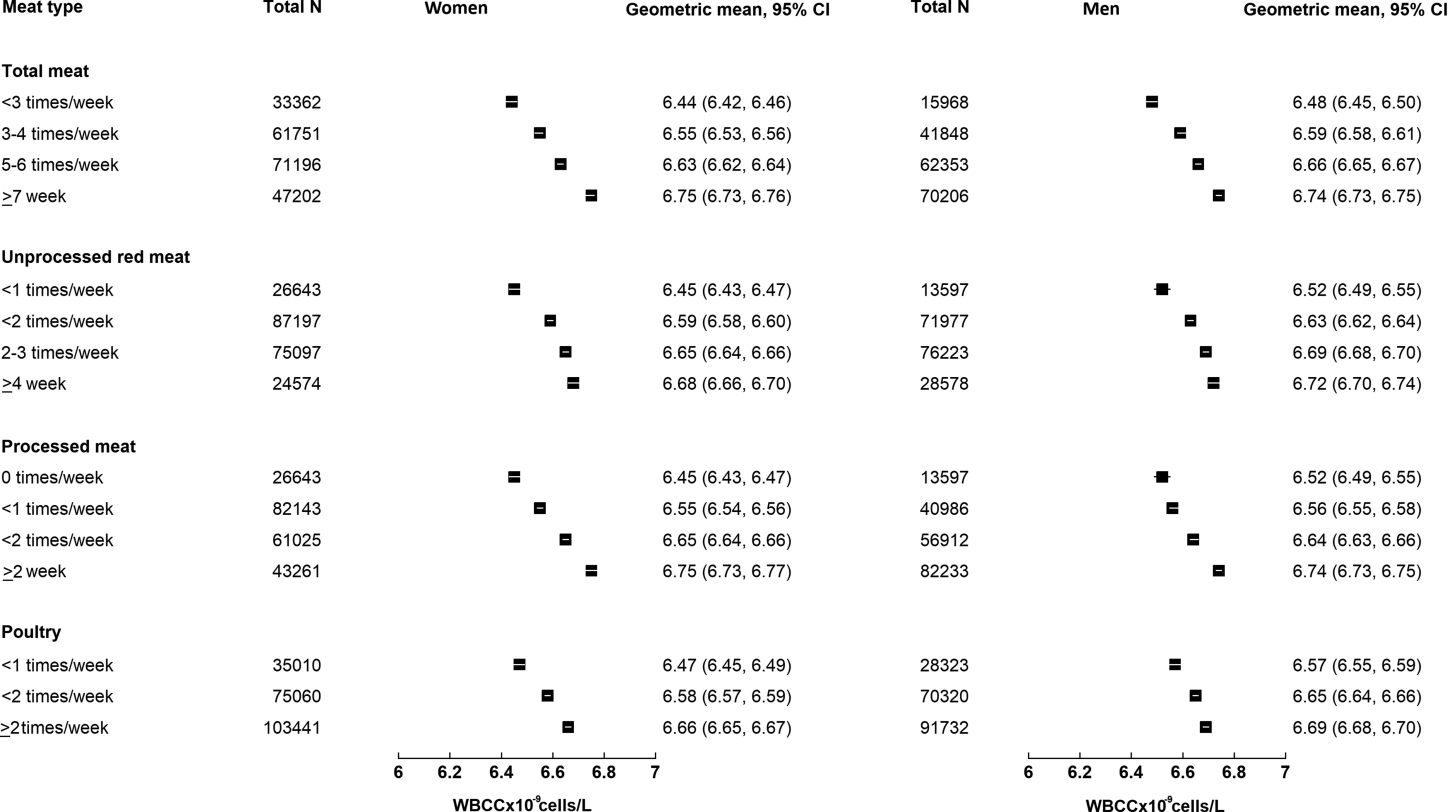
Adjusted geometric means of WBBC (×10^9^ cells/L) and 95% CI by meat types and sex. Adjusted for age, baseline smoking status (never, former, current smoker <15 cigarettes/d, ≥15 cigarettes/d, unknown amount), ethnicity (white, nonwhite), Townsend deprivation index (quintiles from least to most deprived), employment (employed or self-employed, retired, unemployed) and qualification level (college or university degree or vocational qualification, national examination at age 17–18 y, national examination at age 16 y, other or unknown), total fruit and vegetable intake (<3, 3–3.99, 4–5.99, 6+ servings/d), bread and cereal fiber intake (sex-specific quintiles), total fish consumption (0–1, >1 to <2, 2 to <3, ≥3 times/wk), total physical activity (<5, 5–9.9, 10–14.9, 15–24.9, 25–34.9, 35–49.9, 50–74.9, 75–99.9, ≥100 MET h/wk), alcohol intake (<1, 1 to <5, 5 to <10, 10 to <15, 15 to <20, 20 to <25, ≥25, nondrinkers) and menopausal status (premenopausal, postmenopausal) in women. MET, metabolic equivalent; WBCC, white blood cell count.

Each additional 50 g/d intake of meat was associated with higher WBCC. The difference in the WBCC (×10^–9^L) for each 50 g/d higher intake of total meat was 1.5% (95% CI: 1.4, 1.6%), of processed meat was 6.5% (95% CI: 6.1, 6.9%), of unprocessed red meat was 1.6% (95% CI: 1.4, 1.7%), and of poultry was 1.6% (95% CI: 1.4, 1.7%) (Table 2). There were significant interactions by sex for all associations with WBCC (*P* < 0.001 for total meat, processed meat, and poultry and 0.041 for unprocessed red meat). In stratified results both women and men showed a positive association, with larger associations observed in women [difference in the WBCC (×10^–9^L) for each 50 g/d higher intake of total meat in women: 1.7% (95% CI: 1.6,1.9%), in men: 1.2% (95% CI:1.1,1.4%); see Table 2 for meat subtypes]. **Figure 2** shows geometric means for WBCC in women and men by categories of meat intakes (based on model 2).

When additionally adjusting for BMI or waist circumference, we observed similar magnitudes of attenuation for the 2 measures of adiposity. For total meat, the associations were attenuated by 67% for BMI and 58% for waist circumference for CRP, and by 53% and 47% for WBCC based on the estimates (see Table 2 for estimates by subtypes).

In sensitivity analyses using biomarkers at follow-up in a subsample with follow-up biomarker data, baseline intakes of total, unprocessed red, processed meat, and poultry were all positively associated with CRP at follow-up. Similarly, baseline intakes of total, processed meat, and poultry were also positively associated with WBCC at follow-up. However, there was a difference in effect estimates and attenuation with smaller % differences in CRP per 50 g/d intake of processed meat and larger effect estimates for total and unprocessed red meat and poultry intake before and after adjustment for adiposity, and smaller percentage differences in WBCC per 50 g/d intake for unprocessed red meat but little differences for other meat types (**Supplemental Table 1**).

## Discussion

Overall, we found positive associations between any meat intake and 2 inflammatory markers, with larger magnitudes of associations for processed meat, and in women, in this large study of British adults.

Our findings are in line with several previous studies that found small positive associations between red meat (9–13), processed meat (11, 12, 15), and CRP. Previous studies have not found associations between poultry intake and inflammatory markers (15), and to our knowledge no previous studies have investigated associations between meat intake and WBCC.

In most previous studies, adjustment for adiposity attenuated the associations to null (10–12). This was not the case in the present study where associations attenuated substantially (>50%) but remained statistically significant; this might be related to the large size and therefore high power of our study. The remaining associations between meat and inflammatory markers were relatively small (ranging from 0.6 to 15.9% for CRP, mg/L and from 0.3^–9^% to 4.9% for WBCC, × 10  cells/L) and could have been due to residual confounding by other aspects of adiposity such as time exposed to excess weight. In comparison, associations for other lifestyle factors (such as smoking) have been estimated to be around twice as large as what we observed for meat after adjustment for adiposity (19, 20).

Our study findings support the hypothesis that increased adiposity might play a principal role in the association between meat intake, CRP, and WBCC. However, there might also be some independent effects, due for example to meat's heme iron content (4, 21), high saturated fat content (5, 22), and/or AGEs (23), which have each been suggested to be associated with inflammation (mostly assessed by measuring CRP), but none of these putative mediating effects are established.

To our knowledge, this is the largest investigation of habitual meat intake and markers of inflammation to date, but this study has some limitations. UK Biobank study participants are not representative of the UK general population, with UK Biobank participants showing more favorable health behaviors (24). This selection bias could have led to reduced variation in meat intake and inflammatory markers, with those with the least favorable conditions such as very high meat intake and BMI potentially missing from the sample and the results described potentially underestimating a real association. Additionally, UK Biobank did not measure other inflammatory markers (e.g., IL-6 and TNF-α), so these could not be considered in the present study. Moreover, information on some potential confounders was not available, for instance presence of acute infection or details on fasting or nonfasting status. Therefore, there may be residual confounding by these factors (25, 26). Another limitation was the method of dietary assessment; the touchscreen questionnaire did not allow the calculation of total dietary intake to control for potential over- or underreporting. We attempted to account for other dietary factors by adjusting for intakes of total fruit, vegetable, and cereal fiber and of fish, but residual confounding by other aspects of the diet could still operate (27). Moreover, information on diet and adiposity was collected at the same time point. As a result we could not conduct a formal mediation analysis. Future work in this area could assess if the proportion of the association that is attributed to adiposity differs when conducting a mediation analysis (27). The main analysis was cross-sectional, and therefore we cannot assess temporality in all participants; however, we found that most of the associations were similar in a prospective sensitivity analysis in a subsample with follow-up biomarker data.

In this study of British adults, higher meat consumption, particularly of processed meat, was positively associated with inflammatory markers. However, the magnitudes of the associations are small and predominantly due to higher adiposity, and the modest associations remaining after adjustment may be due to residual confounding by other aspects of adiposity.

## Supplementary Material

nxab314_Supplemental_File

## Data Availability

The data, codebook and analytic code described in the manuscript will be made available for bona fide researchers who apply to use the UK Biobank data set by registering and applying at http://www.ukbiobank.ac.uk/register-apply.

## Notes

This work was supported by the Wellcome Trust; Our Planet Our Health; Livestock, Environment and People - LEAP (grant number 205212/Z/16/Z); and the UK Medical Research Council (grant number MR/M012190/1). TYNT was supported by a Nuffield Department of Population Health Intermediate Fellowship; LH was supported by a Lord Crewe fellowship of Lincoln College Oxford. The funders had no role in the design, analysis, or writing of this article.

KP and LH are joint first authors.

Author disclosures: The authors report no conflicts of interest.

This research was conducted using the UK Biobank (UKB) resource under application number 24494.

Supplemental Figure 1 and Supplemental Table 1 are available from the “Supplementary data” link in the online posting of the article and from the same link in the online table of contents at https://academic.oup.com/jn/.

Abbreviations used: AGE, advanced glycation end product; CRP, C-reactive protein; IL-6, interleukin 6; TNF-α, Tumour Necrosis Factor alpha; WBCC, white blood cell count
