# Supplementary material for: Higher Meat Intake Is Associated with Higher Inflammatory Markers, Mostly Due to Adiposity: Results from UK Biobank
Source: J Nutr. 2021 Sep 29;152(1):183–9. doi: 10.1093/jn/nxab314 (PMC8754571; doi:10.1093/jn/nxab314)
Supplement: nxab314_Supplemental_File [file nxab314_supplemental_file.docx]

Table of contents

[Supplemental Table 1 Mean percentage difference (95% CI) in serum CRP and WBCC per 50 g/day higher meat intake by level of adjustment (n=15,420) 2](#_Toc79414434)

[Supplemental Figure 1 Participant flow chart 4](#_Toc79414435)

Supplemental Table 1 Mean percentage difference (95% CI) in serum CRP and WBCC per 50 g/day higher meat intake by level of adjustment (n=15,420)

| **Meat type** | **Model 1**^1^ | | | **Model 2**^2^ | | | **Model 2 + BMI^3^** | | | **Model 2 + WC^4^** | | |
| --- | --- | --- | --- | --- | --- | --- | --- | --- | --- | --- | --- | --- |
| per 50g/day | % difference | 95% CI | p_het_^5^ | % difference | 95% CI | p_het_^5^ | % difference | 95% CI | p_het_^5^ | % difference | 95% CI | p_het_^5^ |
| Serum CRP (mg/L) | | | | | | | | | | | | |
| Total meat^6^ | 14.1 | 11.9,16.5 | 0.270 | 12.5 | 10.3,14.8 | 0.117 | 6.1 | 4.1,8.1 | 0.389 | 7.2 | 5.2,9.3 | 0.096 |
| Women | 15.5 | 12.1,18.9 |  | 14.6 | 11.2,18.0 |  | 6.1 | 3.2,9.0 |  | 8.0 | 5.0,11.1 |  |
| Men | 12.9 | 9.9,16.0 |  | 10.6 | 7.6,13.7 |  | 6.1 | 3.3,9.0 |  | 6.3 | 3.5,9.2 |  |
| Unprocessed red meat^6^ | 19.6 | 15.7,23.7 | 0.739 | 16.6 | 12.7,20.5 | 0.828 | 8.9 | 5.5,12.4 | 0.999 | 10.2 | 6.7,13.7 | 0.588 |
| Women | 18.7 | 13.0,24.8 |  | 17.5 | 11.8,23.6 |  | 8.3 | 3.4,13.4 |  | 10.4 | 5.4,15.7 |  |
| Men | 20.3 | 15.1,25.8 |  | 15.4 | 10.4,20.7 |  | 9.5 | 4.8,14.4 |  | 9.8 | 5.1,14.7 |  |
| Processed meat^6^ | 42.5 | 31.1,55.0 | 0.052 | 30.7 | 20.3,42.1 | 0.014 | 11.7 | 3.2,20.9 | 0.293 | 11.5 | 2.9,20.7 | 0.070 |
| Women | 59.5 | 38.4,83.9 |  | 51.0 | 30.9,74.0 |  | 15.5 | 1.2,31.7 |  | 18.9 | 4.1,35.8 |  |
| Men | 34.0 | 21.0,48.5 |  | 19.9 | 8.2,32.8 |  | 9.5 | -0.9,21.0 |  | 7.3 | -2.9,18.6 |  |
| Poultry^6^ | 14.9 | 10.9,19.0 | 0.018 | 14.7 | 10.7,18.8 | 0.032 | 6.3 | 2.9,9.9 | 0.172 | 8.5 | 5.0,12.2 | 0.060 |
| Women | 20.0 | 14.1,26.3 |  | 18.9 | 13.0,25.1 |  | 7.0 | 2.1,12.2 |  | 10.2 | 5.1,15.5 |  |
| Men | 10.0 | 4.8,15.5 |  | 10.9 | 5.7,16.4 |  | 5.6 | 0.8,10.7 |  | 6.6 | 1.7,11.7 |  |
| WBCC (x10^9^ cells/L) | | | | | | | | | | | | |
| Total meat^6^ | 1.6 | 1.0,2.2 | 0.298 | 1.4 | 0.8,2.0 | 0.454 | 0.8 | 0.2,1.4 | 0.306 | 0.9 | 0.3,1.5 | 0.455 |
| Women | 1.3 | 0.3,2.2 |  | 1.3 | 0.3,2.2 |  | 0.5 | -0.4,1.5 |  | 0.7 | -0.3,1.6 |  |
| Men | 1.9 | 1.1,2.7 |  | 1.6 | 0.8,2.4 |  | 1.1 | 0.3,1.9 |  | 1.1 | 0.3,1.9 |  |
| Unprocessed red meat^6^ | 1.2 | 0.3,2.2 | 0.045 | 0.9 | -0.1,1.9 | 0.215 | 0.2 | ,0.8,1.2 | 0.185 | 0.3 | -0.7,1.3 | 0.257 |
| Women | 0.3 | -1.3,1.8 |  | 0.3 | ,1.2,1.9 |  | ,0.5 | -2.0,1.1 |  | ,0.3 | -1.9,1.2 |  |
| Men | 2.1 | 0.9,3.4 |  | 1.4 | 0.1,2.7 |  | 0.7 | -0.5,2.0 |  | 0.8 | -0.5,2.0 |  |
| Processed meat^6^ | 9.1 | 6.4,11.8 | 0.085 | 6.8 | 4.2,9.5 | 0.106 | 5.1 | 2.5,7.7 | 0.031 | 5.0 | 2.4,7.6 | 0.057 |
| Women | 5.8 | 1.2,10.6 |  | 3.8 | -0.7,8.5 |  | 1.1 | -3.3,5.7 |  | 1.3 | -3.1,5.9 |  |
| Men | 10.9 | 7.8,14.1 |  | 8.7 | 5.6,11.8 |  | 7.5 | 4.5,10.6 |  | 7.2 | 4.2,10.3 |  |
| Poultry^6^ | 1.8 | 0.8,2.9 | 0.096 | 2.1 | 1.0,3.1 | 0.264 | 1.3 | 0.2,2.3 | 0.412 | 1.5 | 0.4,2.5 | 0.322 |
| Women | 2.7 | 1.1,4.4 |  | 2.9 | 1.3,4.6 |  | 1.9 | 0.2,3.5 |  | 2.1 | 0.5,3.8 |  |
| Men | 1.1 | -0.3,2.4 |  | 1.4 | 0.1,2.8 |  | 0.8 | -0.5,2.2 |  | 1.0 | -0.4,2.3 |  |

CI, confidence interval; CRP, C-reactive protein; BMI, body mass index; WBCC, white blood cell count; WC, waist circumference;

The percent difference refers to an increase of in CRP/WBCC of every 50g/day higher meat intake.

^1^Model 1 adjusted for age. ^2^Model 2: Model 1 + baseline smoking status (never, former, current smoker <15 cigarettes/day, ≥15 cigarettes/day, unknown amount), ethnicity (white, non-white), Townsend deprivation index (quintiles from least to most deprived), employment (employed or self-employed, retired, unemployed) and qualification level (college or university degree or vocational qualification, national examination at ages 17-18, national examination at age 16, other or unknown), total fruit and vegetable intake (<3, 3-3.99, 4-5.99, >6 servings per day), bread and cereal fiber intake (sex-specific quintiles), total fish consumption (0-1,, >1-<2, 2-<3, >3 times per week), total physical activity (<5, 5-9.9, 10-14.9, 15-24.9, 25-34.9, 35-49.9, 50-74.9, 75-99.9, ≥100 metabolic equivalent hours per week), alcohol intake (<1, 1 <5, 5 <10, 10 <15, 15 <20, 20 <25, ≥25, Non-drinkers) and menopausal status (pre-menopausal/post-menopausal) in women. ^3^Model 2 + baseline body mass index (continuous). ^4^Model 2 + baseline waist circumference (continuous). ^5^P heterogeneity based on an LR test comparing the model with and without an interaction for sex. ^6^% change BMI/WC is the proportion of the main association (Model 2) attenuated after adjustment for adiposity. ^6^Association for women and men combined, all models additionally adjusted for sex.

Basic exclusion criteria:

- Withdrawn consent (n=829)

Analysis related exclusion criteria:

- Missing information for CRP and WBCC (n=45,965)
- Missing information for meat intake (n=6806)
- Missing information for covariates (n=45,831)

**403,886 maximal analytical sample**

502,488 participants

503,317 participants recruited

(2006-2010)

Supplemental Figure 1 Participant flow chart
